# Local administration of siRNA through Microneedle: Optimization, Bio-distribution, Tumor Suppression and Toxicity

**DOI:** 10.1038/srep30430

**Published:** 2016-07-26

**Authors:** Tao Tang, Yan Deng, Jiao Chen, Yi Zhao, Ruifeng Yue, Kwong Wai Choy, Chi Chiu Wang, Quan Du, Yan Xu, Linxiao Han, Tony Kwok Hung Chung

**Affiliations:** 1 Department of Obstetrics & Gynaecology, Faculty of Medicine, The Chinese University of Hong Kong, Shatin, New Territories, Hong Kong, China; 2 Shenzhen Research Institute, The Chinese University of Hong Kong, China; 3 Institute of Microelectronics, Tsinghua University, Beijing, China; 4 Li Ka Shing Institute of Health Sciences, Faculty of Medicine, The Chinese University of Hong Kong, Shatin, New Territories, Hong Kong, China; 5 School of Biomedical Sciences, Faculty of Medicine, The Chinese University of Hong Kong, Shatin, New Territories, Hong Kong, China; 6 State Key Laboratory of Natural and Biomimetic Drugs, School of Pharmaceutical Sciences, Peking University, Beijing, China; 7 Department of Obstetrics & Gynaecology, Guangzhou panyu Central hospital, Guangzhou, China; 8 Dongguan Third People’s Hospital, Affiliated Dongguan Shilong People’s Hospital of Southern Medical University, Dongguan, China

## Abstract

Although RNA interference may become a novel therapeutic approach for cancer treatment, target-site accumulation of siRNA to achieve therapeutic dosage will be a major problem. Microneedle represents a better way to deliver siRNAs and we have evaluated for the first time the capability of a silicon microneedle array for delivery of Gapdh siRNA to the skin *in vivo* and the results showed that the microneedle arrays could effectively deliver siRNA to relevant regions of the skin noninvasively. For the further study in this field, we evaluated the efficacy of the injectable microneedle device for local delivery of siRNA to the mouse xenograft. The results presented here indicate that local administration of siRNA through injectable microneedle could effectively deliver siRNA into the tumor region, and inhibit tumor progression without major adverse effects.

RNA interference (RNAi) is one of the most powerful and widely used tools for the study of gene function and has been developed as a novel therapeutic tool to target specific genes for the treatment of diseases, and it is defined as a mechanism of specific post-transcriptional gene-silencing mediated by small RNAs, including endogenous microRNA (miRNA) and exogenous chemical synthesized small interfering RNA (siRNA) or short hairpin RNA (shRNA)^1^. RNAi might become a novel therapeutic approach for cancer treatment because researchers can easily design siRNA molecules to inhibit, specifically and potently, the expression of proteins involved in tumor initiation and progression.

Although siRNA has the potential to be a novel class of drugs, the limited cellular uptake, low biological stability, and unfavorable pharmacokinetics of siRNAs have hampered their application in the clinic^2^. When using systemic delivery strategies, nucleic acids in general are exposed to nuclease degradation, which makes systemic RNAi delivery more complicated and challenging. In addition, studies show that systemically administered naked siRNA preferentially accumulated in kidney and excreted into urine within 1 h due to its relatively small size compared to the effective glomerular pore size. As a result, target-site accumulation of siRNA to achieve therapeutic dosage will be a major problem^3^,^4^,^5^. Therefore, the translation of siRNA to the clinical setting is highly dependent on the development of an appropriate delivery system, which is able to ameliorate the pharmacokinetic and bio-distribution properties of siRNA.

Microneedle represents a better way to deliver siRNAs, as it has been shown to penetrate the skin, across the *stratum corneum*, and into the viable epidermis. It can avoid direct contact with blood vessels and nerve fibers which reside in the dermal layer. As a result, the major benefit of using microneedle is pain free^6^. There are 2 kinds of microneedles< solid and hollow. Solid microneedles can be used as skin pretreatment. By inserting the microneedles to form pores, a drug formulation can be applied to the skin for slow diffusion through the pores and into the body; while hollow microneedles can be used for infusion of liquid formulations into the skin through the needle hole^7^.

In previous study, we evaluated for the first time the capability of a silicon microneedle array for delivery of the housekeeping gene (Gapdh) siRNA to the skin of the mouse *in vivo* and the results showed the microneedle arrays could effectively deliver siRNA to relevant regions of the skin noninvasively^8^. For the further proof-of-concept study in this field, we evaluated the efficacy of the injectable microneedle device for local deliver of siRNA to the mouse xenograft. The bio-distribution, tumor suppression activity and side effects of siRNA were also tested.

## Results

### Injectable microneedle device

**Figure 1:**
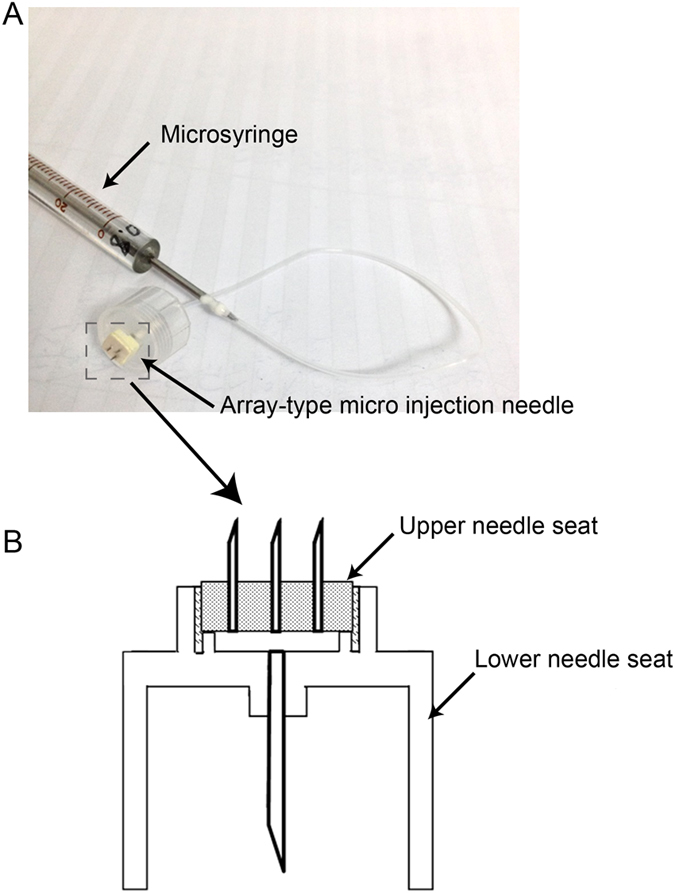
The image of injectable microneedle device. (**A**) It consists of a micro-syringe, an array-type micro injection needle head and a tube connecting them. (**B**) Structure of the array-type micro injection needle head.

Figure 1 is the view of the injectable microneedle device, in which, the array-type micro- injection needle head comprises a lower needle seat. It was configured as a cylindrical column opening at one end and comprising a top cap at the other end, and an upper needle seat located over the top cap of the lower needle seat with a cavity (100 μm in height) formed between the upper needle seat and the top cap. The top cap was provided with a through-hole for communicating the cylindrical column with the cavity, and a pipetting needle (304 25G stainless steel needle) was positioned in the through-hole, connecting to the syringe by tube. Three hollow needles (304 33G stainless steel needle) were mounted in the upper needle seat, arranged in an equilateral triangle shape. For each, the bevel angle of the tip was 30 degree and was fabricated with 500 or 800 μm in height. The distance between the two needles was 1.5 mm.

### Knockdown efficiency and tumor suppressing effect of Human papillomavirus (HPV) 16 E6 siRNA delivered by the microneedle in cervical tumor xenograft

To investigate the therapeutic effect of siRNA delivered by injectable microneedle into the cervical cancer, we chose siRNA which specifically and effectively silences HPV16 E6 oncogene. This gene has received lots of attention, as E6 oncoprotein interacts with several cellular proteins, thereby activating a number of oncogenic pathways that lead to blockage of apoptosis, alterations of the transcription machinery, interference with cell-cell interactions and cell immortalization in the pathogenesis of cervical cancer^9^. To assess the effect of silencing E6 gene on the growth of SiHa cells, we have performed *in vitro* MTT assay and qRT-PCR. Noticeably, we observed significant reduction in the E6 gene expression and the proliferation of SiHa cells treated with HPV16 E6 siRNA (Supplementary Figs S1 and S2). In this study, we adopted the xenograft mouse models of cervical cancer.

**Figure 2:**
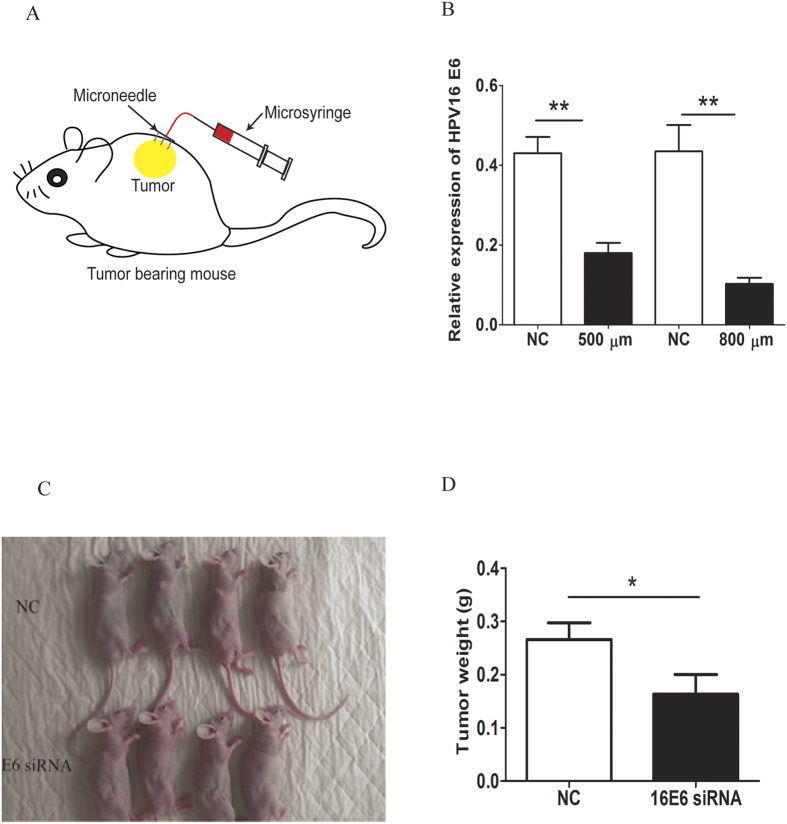
(**A**) Methods of drug delivery to the skin using injectable microneedle device; (**B**) HPV16 E6 gene expression detected in the tumor treated with the siRNA delivered by either 500 μm or 800 μm 24 h after administration. A marked reduction of HPV16 E6 gene expression was detected in the tumor treated with the siRNA delivered by either 500 μm or 800 μm 24 h after administration. The 800 μm injectable microneedle showed higher knockdown efficiency. (**C**) Ten days post first treament, SiHa xenografts were photographed. (**D**) Statistical analysis for tumor weight of SiHa xenografts. Significant suppression of tumor growth in animals treated with E6 siRNA was observed. NC stands for nonsense siRNA. T test, *P < 0.05.

In our *in vitro* data (Supplementary Fig. S1), we chose 24 h as the time point for gene knockdown studies (qRT-PCR) and significant silencing effect could be observed at this time point, which was also confirmed in our previous study^8^. To be consistent with the *in vitro* study, we chose 24 h for the *in vivo* measurement as well. Two kinds of injectable microneedles were fabricated with 500 μm or 800 μm in height and connected to a micro-syringe. A marked reduction of HPV16 E6 gene expression was detected in the tumor treated with the siRNA delivered by either 500 μm or 800 μm 24 h after administration. The 800 μm injectable microneedle showed higher knockdown efficiency (Fig. 2B). As a result, we used this type of needle for injection in the following study. 5′ cholesterol HPV16 E6 siRNA was administered locally through 800 μm injectable microneedle. Animals that received non-sense control (NC) siRNA were used as control to assess the extent of tumor suppression. We observed significant suppression of tumor growth in animals treated with E6 siRNA (*P* < 0.05) (Fig. 2C,D).

### Distribution of HPV16 E6 siRNA delivered by the microneedle in cervical tumor xenograft

**Figure 3:**
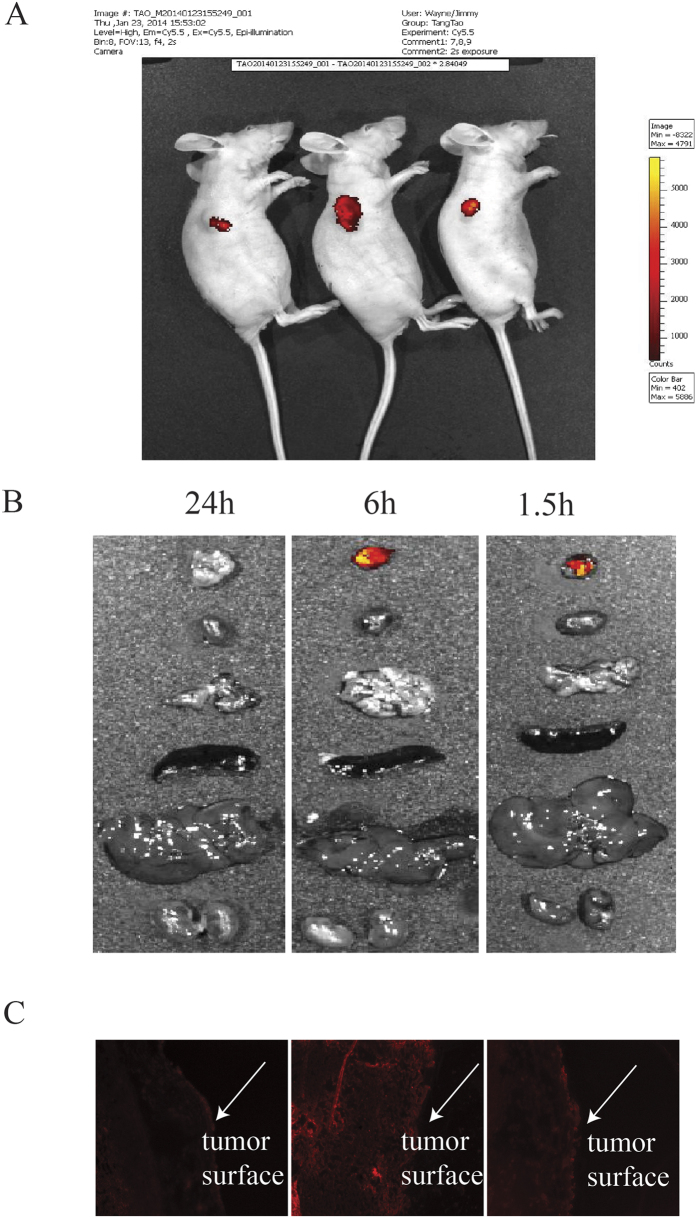
Cy5-labeled cholesterol siRNA distribution in both organ and tissue level. (**A**) The fluorescence intensity in the whole body and (**B**) dissected tumor, heart, lung, spleen, liver and kidney; (**C**) The fluorescence intensity in the tumor tissue section.

Nine mice were treated with cy5-labeled human HPV16 E6 siRNA. The distribution of the siRNAs in both organ and tissue levels were visualized by the biophotonic *in vivo* imaging analysis (IVIS 200 imaging system) and the siRNA florescence histology analysis (fluorescence microscope) was performed at the time points of 1.5 h, 6 h, 24 h after treatment. As shown in Fig. 3, the fluorescence intensity was strong in the tumor and absent in the heart, lung, spleen, liver and kidney at the time point of 1.5 h and 6 h. However, there was almost no fluorescent signal observed in the tumor and no fluorescent signal was observed in other organs at the time point of 24 h. Subsequently, the distribution of the Cy5-labeled siRNA within the tumor was examined after the experiment. The tumor was dissected and embedded in optimal cutting temperature (OCT) compound for cryosection and microscopic examination. At the time of 1.5 h, Cy5-labeled siRNA mainly accumulated under the skin; at 6 h, it penetrated into the tumor and 24 h later the signal quenched.

### Adverse effects of the HPV16 E6 siRNA

**Figure 4:**
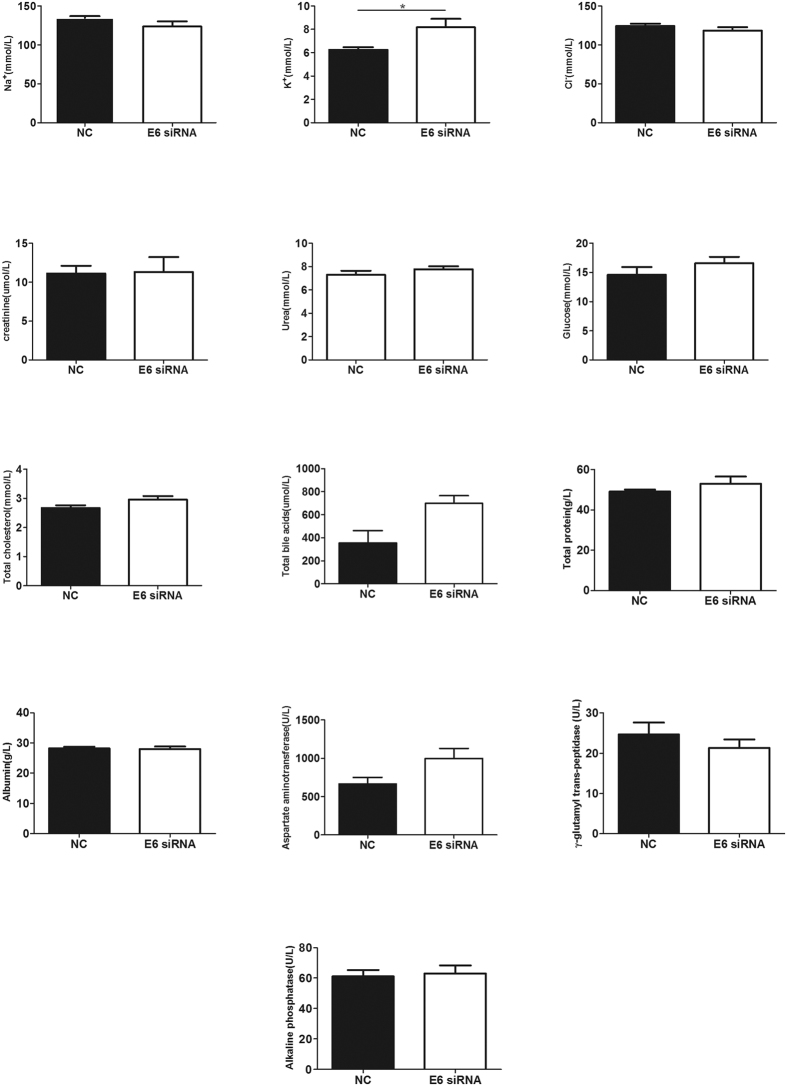
Biochemistry analysis of blood taken from mice including including sodium, potassium, chloridion, glucose, total cholesterol, urea, creatinine, total protein and albumin, aspartate aminotransferase, alkaline phosphatase, γ-glutamyl trans-peptidase and bile acids. A significant increase in the concentration of potassium iron was observed. NC stands for nonsense siRNA. T test, *P < 0.05.

We have also collected the blood samples from the mice for the biochemistry analysis to investigate the major toxic effects of the HPV16 E6 siRNA. Sodium, potassium and chloride ion are the representatives of electrolyte. Creatinine and urea are the important indicators of renal health. Total cholesterol, total protein and albumin, aspartate aminotransferase, alkaline phosphatase, γ-glutamyl transpeptidase and bile acids are indicators of hepatocellular effects. The results of the blood biochemical tests are shown in Fig. 4. Compared with the NC group, minor changes were observed in the HPV16 E6 siRNA group, but it caused a significant increase in the concentration of potassium ion (*P* < 0.05). The treatment also showed a trend to increase bile acid, aspartate aminotransferase, and γ-glutamyl transpeptidase and decrease alkaline phosphatase, although there was no statistical difference.

## Discussion

SiRNA induced gene silencing mainly achieved by blocking their targets’ function hijacks the inhibitory effects of conventional drugs. However, some disease-related molecules are considered as “non-druggable” targets. Up to date, many of these have been targeted by siRNA approach and new siRNA-based drugs have been developed for clinical trials^3^,^10^. Cancer is a genetic disease which involves mutational and epigenetic changes which lead to uncontrollable cell proliferation and differentiation^11^,^12^. SiRNA have been extensively used to silence cancer-related gene targets. A line of preclinical studies have suggested that gene silencing can inhibit cancer development including tumor growth, angiogenesis, metastasis and chemo-resistance^13^,^14^,^15^. From a clinical perspective, siRNA approach may represent a more suitable and safe strategy for the management of cervical cancer when compared to commonly-used radiation or chemo-radiation therapy. Since cervical cancer is mainly caused by HPV, and HPV is the most important etiological agent. Importantly, HPV oncogene is exogenous. Therefore, siRNA treatment would not affect the host genes. On the other hand, for the Stage 0 and Stage I cervical cancer, abnormal cells/a very small amount cancer cells are found only in the innermost tissue of cervix and local treatment may be enough and appropriate for the patients. As a result, the cervical cancer model is ideal for testing the anti-cancer effects of siRNA locally delivered by microneedles.

For the siRNA application, the major challenge to clinical exploitation is lack of suitable and effective delivery tools^16^. The siRNA has been delivered through different systems for animal studies, including systemic delivery through the intravenous injection, or local delivery like intratumoral administration^17^. In previous study, hypodermic needles were used for delivery of therapeutic siRNA, which caused significant pain. To provide a less invasive method, microneedle devices were investigated^18^,^19^. In a pilot study, we have evaluated the ability of a silicon microneedle array to punch holes for delivery of the housekeeping gene (*Gapdh*) siRNA to mouse ear skin and found that it could effectively deliver siRNA to relevant regions of the skin with efficiency^8^. Subsequently, we have compared the differences between intratumoral injection by silicon microneedle array and normal syringe. It seems that microneedle-based siRNA delivery protocol significantly reduced the siRNA distribution in the other organs in mice (Supplementary Fig. S3). While, in this study, we adopted hollow injectable microneedle and the advantage is that hollow microneedle is designed for fast and efficient delivery of liquid medication. It can improve skin permeability, make the dose accurate and enhance “patient” compliance.

By using the hollow injectable microneedle, the distribution of siRNA in the tumor region and major organs after administration was detected by investigating the distribution of fluorescence. In previous study, we used biophotonic imaging technology to examine the organ distribution of FAM-labeled siRNA (delivered by the liposome or as free siRNA) after *in vivo* systemic administration. The intensity of the targeting region fluorescence signal was the strongest. However, the fluorescence signal was also detectable in the heart, spleen, lungs and kidneys of the rats from all of the treatment groups^20^. Compared with systemic administration, local delivery of siRNA solution might maintain therapeutic concentration in the local region, the fluorescence intensity was strong in the tumor but absent in the heart, lung, spleen, liver and kidney at the time point of 1.5 h and 6 h. Also, a small volume of siRNA solution might be suitable for maintaining therapeutic concentration in the specific region. Compared to NC group, a marked reduction of HPV16 E6 gene expression was detected in the tumor treated with the siRNA 24 h after administration, although there seems no fluorescence intensity observed at that time point. One of the possible reasons is that the 3′end of the siRNA sense strand was modified by the fluorescent dye cy5. When siRNA enters the RNAi pathway, the anti-sense strand would be loaded into the (RNA-induced silencing complex) RISC to become the guide strand for targeting and degrading mRNA and the sense strand was cleaved. As a result, the silencing effect could be observed although the fluorescence was weak or disappeared at 24 h post delivery. When comparing the mice at different time points, the fluorescent signal was strong and observed moderately distributed in the epidermis near the deposited “plug” 1.5 h post administration. In contrast, the red signal at 6 h post administration was widely distributed and weaker than that of 1.5 h, which suggesting uptake and cytoplasmic localization of the siRNAs. It may also due to the limit of IVIS 200 imaging system for deep scanning. At 24 h, little signals were observed. This may be the remaining siRNA “trapped”between the epidermis and tumor, which did not enter the RNAi pathway. We also observed significant suppression of tumor growth in animals treated with E6 siRNA every three days. This result showed that the effect of tumor suppression induced by local delivery of E6 siRNA which silenced the HPV16 E6 gene.

In addition to the current enthusiasm around RNA interference as an emerging novel approach to treat human diseases, a key challenge of this technology is the safe to the body. However, there is little information regarding to the safety of locally administered siRNA. A recent study suggested that local administration (direct intra-tumorous injection) of Nek2 siRNA is a safe delivery procedure with little cytotoxicity^19^. In our study, the results of the blood biochemical tests indicated that local administration of siRNA cause few adverse effects, only showing a trend to increase bile acid, aspartate aminotransferase, and γ-glutamyl transpeptidase and decrease alkaline phosphatase with no statistical difference. However, further investigation including an orthodox preclinical toxicology study is needed for the potential clinical application. As an orthodox program, it typically includes selection of a rodent species and a non-rodent species (such as rat and monkey). In addition, single-dose and repeat-dose comparative tolerability studies should be included to determine the maximum tolerated dose and the no observable adverse effect level^21^. Then the safety profile can be translated to humans.

In conclusion, the results presented here indicate that local administration of siRNA through injectable microneedle could effectively deliver siRNA into the tumor region, and inhibit tumor progression without major adverse effects. Referring to translation to clinical application, it may provide an alternative to the conventional approaches.

## Materials and Methods

### Injectable microneedle device

The device consists of a micro-syringe, an array-type micro injection needle head and a tube connecting them.

### Animals

Experiments were performed on female Balb/c athymic mouse (6 weeks old) bred and kept by the Laboratory Animal Services Center of the Chinese University of Hong Kong. The methods were carried out in accordance with the approved guidelines.

### Ethics statement

All experimental protocols were approved by the Animal Experimentation Ethics Committee of the Chinese University of Hong Kong and the Government of the Hong Kong SAR.

### SiRNAs

The 3′ labeled cy5 and 5′ cholesterol human HPV16 E6 siRNA, 5′ cholesterol human HPV16 E6 siRNA, 5′ cholesterol nonsense siRNA and nonsense siRNA were purchased from RioboBio (Guangzhou, China).

### Cell culture

Cervical cancer cell line SiHa (American Type Cell Culture Collection, VA, USA) was cultured in Dulbecco’s modified Eagle’s medium (DMEM, Hyclone, GE Health Care, USA) supplemented with 10% fetal bovine serum. Cells were incubated in a humidified atmosphere with 5% CO_2_ at 37 °C.

### Examination of knockdown efficiency of HPV16 E6 siRNA delivered by the microneedle in cervical tumor xenograft mice model

Twenty Balb/c athymic mice were anesthetized by Ketamine (75 mg/kg, i.p.)/Xylazine (10 mg/kg, i.p.) and then cervical cancer cells (SiHa, 2 × 10^6^ cells) were injected into the back flank of mice by subcutaneous injection using micro-syringe fitted with a 30G needle. Fourteen days post tumor inoculation, the animals were subjected to the following treatments: 1) human HPV16 E6 siRNA and n = 5 for each type of microneedles (500 and 800 μm); 2) Nonsense siRNA group as control (NC group, n = 5 for each type). And 5 μl of the siRNA solution (10 μg/μl) was directly intra-tumorous injected with microneedle (Fig. 2A). The knockdown efficiency of the siRNA was detected by gene expression analysis using qRT-PCR (compared with nonsense siRNA group) at time point of 24 h after treatment. The microneedle with higher knockdown efficiency was used for the following experiments.

### Analysis of gene silencing

Mice were euthanized at 24 h after siRNA treatment and the tumor was dissected for analysis by qRT-PCR as follows with the nonsense siRNA group as control. Total RNA was extracted by homogenization in 1 ml Trizol reagent, followed by chloroform extraction and isopropanol precipitation. 100 ng sample of total RNA was reverse-transcribed to cDNA using the commercial protocol. For mRNA amplification, the primers: 5′-CACAGGAGCGACCCAGAAAG-3′ (E6 forward), 5′-GCATAAATCCCGAAAAGCA A-3′ (E6 reverse) were used. Quantitative Real-time Polymerase Chain Reaction will performed by using Power SYBR^®^ Green PCR Master Mix (Applied Biosystems, Foster City, California). For the analysis of Real-time PCR Data: the relative expression of target mRNA was determined by dividing the target amount by endogenous control amount to obtain a normalized target value. Then the normalized values of the target mRNA were compared among the samples.

### Test of therapeutic effect of HPV16 E6 siRNA delivered by the microneedle in cervical tumor xenograft mice model

Twenty Balb/c athymic mice with cervical tumor xenograft were employed for this approach. The HPV16 E6 siRNA (n = 10) and nonsense siRNA (n = 10, as control) were treated for Balb/c athymic mice with corresponding cervical tumor xenograft by microneedle based delivery system every three days. Ten days after first siRNA treatment, the mice were terminated and the weight of cervical tumor xenograft was measured.

### Examination of the distribution of HPV16 E6 siRNA delivered by the microneedle in cervical tumor xenograft mice model

Another nine mice were treated with cy5 labeled human HPV16 E6 siRNA group and n = 3 for each time point (1.5, 6 and 12 h). The distribution of the siRNAs in both organ and tissue level was visualized by the biophotonic *in vivo* imaging analysis (IVIS 200 imaging system) and the siRNA florescence histology analysis (fluorescence microscope) at time points of 1.5 h, 6 h, 24 h after treatment^22^.

### Test of adverse effect of HPV16 E6 siRNA delivered by the microneedle in cervical tumor xenograft mice model

The blood from the mice was collected for the biochemistry analysis to investigate the major toxic effect of the siRNA, including sodium, potassium, chloride ion, glucose, total cholesterol, urea, creatinine, total protein and albumin, aspartate aminotransferase, alkaline phosphatase, γ-glutamyl trans-peptidase and bile acids.

### Data analysis

Numeric data in each group will be expressed as mean ± standard deviation and analyzed by Analysis of Covariance (ANOVA) with LSD’s post hoc multiple comparisons or unpaired t-test as appropriate. Statistical significance is set at *P* < 0.05.

## Additional Information

**How to cite this article**: Tang, T. *et al*. Local administration of siRNA through Microneedle: Optimization, Bio-distribution, Tumor Suppression and Toxicity. *Sci. Rep*. **6**, 30430; doi: 10.1038/srep30430 (2016).

## Supplementary Material

Supplementary Information

## References

[b1] Wang Z., Rao D. D., Senzer N. & Nemunaitis J. RNA interference and cancer therapy. Pharmaceutical research 28, 2983–2995 (2011). 22009588 10.1007/s11095-011-0604-5

[b2] Ozpolat B., Sood A. K. & Lopez-Berestein G. Liposomal siRNA nanocarriers for cancer therapy. Advanced drug delivery reviews 66, 110–116 (2014). 24384374 10.1016/j.addr.2013.12.008 PMC4527165

[b3] Deng Y. . Therapeutic potentials of gene silencing by RNA interference: principles, challenges, and new strategies. Gene 538, 217–227 (2014). 24406620 10.1016/j.gene.2013.12.019

[b4] Guo P. X. . Engineering RNA for Targeted siRNA Delivery and Medical Application. Advanced drug delivery reviews 62, 650–666 (2010). 20230868 10.1016/j.addr.2010.03.008 PMC2906696

[b5] Hong C. A. & Nam Y. S. Functional Nanostructures for Effective Delivery of Small Interfering RNA Therapeutics. Theranostics 4, 1211–1232 (2014). 25285170 10.7150/thno.8491 PMC4183999

[b6] Hickerson R. P. . Gene Silencing in Skin After Deposition of Self-Delivery siRNA With a Motorized Microneedle Array Device. Mol Ther-Nucl Acids 2 (2013). 10.1038/mtna.2013.56 PMC4027428 24150576

[b7] Kim Y. C., Park J. H. & Prausnitz M. R. Microneedles for drug and vaccine delivery. Advanced drug delivery reviews 64, 1547–1568 (2012). 22575858 10.1016/j.addr.2012.04.005 PMC3419303

[b8] Deng Y. . Transdermal Delivery of siRNA through Microneedle Array. Scientific reports 6 (2016). 10.1038/srep21422 PMC4757825 26888011

[b9] Chakrabarti O. & Krishna S. Molecular interactions of ‘high risk’ human papillomaviruses E6 and E7 oncoproteins: implications for tumour progression. J Biosciences 28, 337–348 (2003). 10.1007/BF02970152 12734411

[b10] Daka A. & Peer D. RNAi-based nanomedicines for targeted personalized therapy. Advanced drug delivery reviews 64, 1508–1521 (2012). 22975009 10.1016/j.addr.2012.08.014

[b11] McLeod H. L. Cancer Pharmacogenomics: Early Promise, But Concerted Effort Needed. Science 339, 1563–1566 (2013). 23539596 10.1126/science.1234139 PMC3900028

[b12] Suva M. L., Riggi N. & Bernstein B. E. Epigenetic Reprogramming in Cancer. Science 339, 1567–1570 (2013). 10.1126/science.1230184 PMC3821556 23539597

[b13] Zhang W. . B7-H3 silencing by RNAi inhibits tumor progression and enhances chemosensitivity in U937 cells. OncoTargets and therapy 8, 1721–1733 (2015). 26203263 10.2147/OTT.S85272 PMC4508088

[b14] Huang P. I. . Non-Viral Delivery of RNA Interference Targeting Cancer Cells in Cancer Gene Therapy. Curr Gene Ther 12, 275–284 (2012). 22856602 10.2174/156652312802083576

[b15] Ramachandran P. V. & Ignacimuthu S. RNA Interference as a Plausible Anticancer Therapeutic Tool. Asian Pac J Cancer P 13, 2445–2452 (2012). 10.7314/apjcp.2012.13.6.2445 22938402

[b16] Dahlman J. E., Kauffman K. J., Langer R. & Anderson D. G. Nanotechnology for *In vivo* Targeted siRNA Delivery. Adv Genet 88, 37–69 (2014). 25409603 10.1016/B978-0-12-800148-6.00003-1

[b17] Asai T. & Oku N. Systemic Delivery of Small RNA Using Lipid Nanoparticles. Biol Pharm Bull 37, 201–205 (2014). 24492716 10.1248/bpb.13-00744

[b18] Leachman S. A. . First-in-human Mutation-targeted siRNA Phase Ib Trial of an Inherited Skin Disorder. Mol Ther 18, 442–446 (2010). 19935778 10.1038/mt.2009.273 PMC2839285

[b19] Kokuryo T., Yokoyama Y. & Nagino M. The analysis of the transmission rate of locally administered siRNA to blood. Faseb J 27 (2013).

[b20] Zhang G. . A delivery system targeting bone formation surfaces to facilitate RNAi-based anabolic therapy. Nature medicine 18, 307–314 (2012). 10.1038/nm.2617 22286306

[b21] Barros S. A. & Gollob J. A. Safety profile of RNAi nanomedicines. Advanced drug delivery reviews 64, 1730–1737 (2012). 22732527 10.1016/j.addr.2012.06.007

[b22] Li S. D., Chen Y. C., Hackett M. J. & Huang L. Tumor-targeted delivery of siRNA by self-assembled nanoparticles. Mol Ther 16, 163–169 (2008). 17923843 10.1038/sj.mt.6300323 PMC2739987

