# Supplementary material for: Local administration of siRNA through Microneedle: Optimization, Bio-distribution, Tumor Suppression and Toxicity
Source: Sci Rep. 2016 Jul 26;6:30430. doi: 10.1038/srep30430 (PMC4960650; doi:10.1038/srep30430)
Supplement: Supplementary Information [file srep30430-s1.pdf]

Supplementary Materials for
Local administration of siRNA through Microneedle: Optimization, Bio-distribution, Tumor Suppression and Toxicity

Authors: Tao TANG *#， Yan DENG#, Jiao CHEN, Yi ZHAO, Ruifeng YUE, Kwongwai CHOY, Chichiu WANG, Quan DU, Yan XU, Linxiao HAN, Tony Kwokhung CHUNG

# The authors contributed equally

*****Corresponding Author: Dr. Tao TANG (tangtao@cuhk.edu.hk)

This file includes:

Supplementary Figure S1. Transfection of HPV16 E6 siRNA strongly inhibited targeted gene expression in cervical cancer cells.

Supplementary Figure S2. Transfection of HPV16 E6 siRNA significantly inhibited the proliferation of cervical cancer cells.

Supplementary Figure S3. Distribution of fluorescence labeled siRNA in the organs.

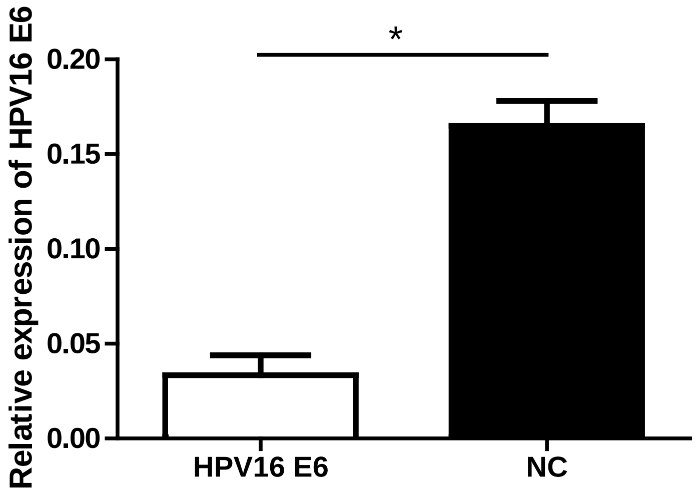

Supplementary Figure S1 Transfection of HPV16 E6 siRNA into cervical cancer cell (SiHa) was performed by using Lipofectamine™ 2000 with nonsense siRNA (NC) as control. Cells were collected 24 h post transfection and qRT-PCR was used for evaluation of the silencing effect for E6. A marked reduction of E6 gene expression was observed (*P<0.05 compared with NC group, t-test).

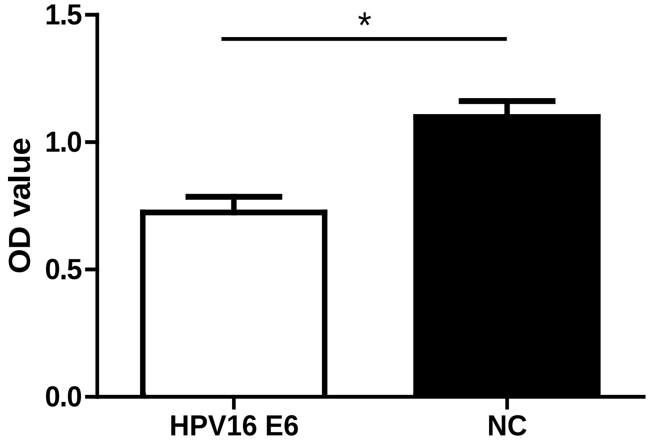

Supplementary Figure S2 Transfection of HPV16 E6 siRNA into cervical cancer cell (SiHa) was performed by using Lipofectamine™ 2000 with nonsense siRNA (NC) as control. Twenty-four hour post transfection, MTT Assay was used for detection of cell viability/proliferation. HPV16 E6 siRNA significantly inhibited the proliferation of SiHa cells (*P<0.05 compared with NC group, t-test).

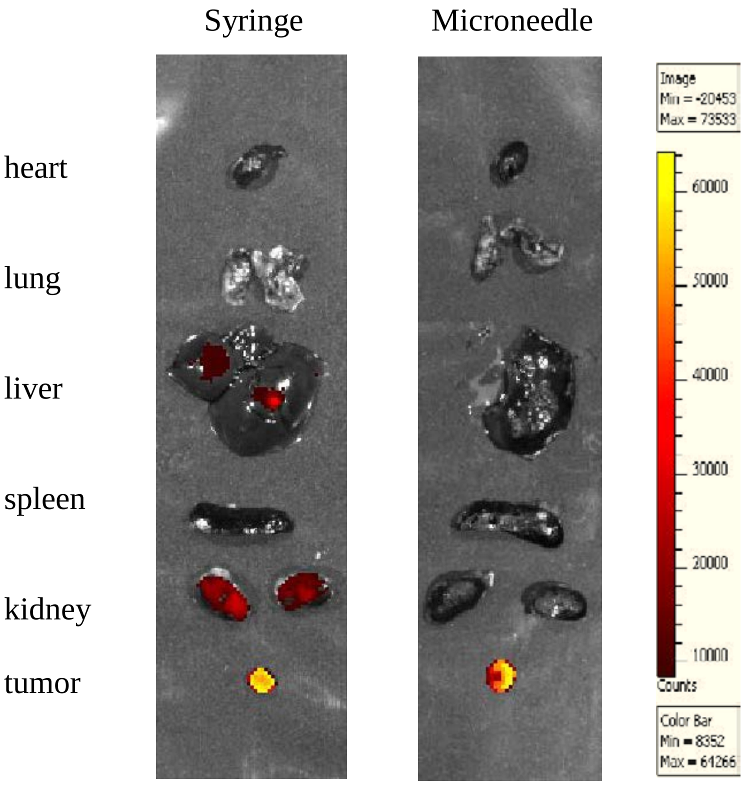

Supplementary Figure S3 Six hour post intratumoral injection, the distribution of fluorescence labeled siRNA was monitored by IVIS 200 imaging system. Microneedle based siRNA delivery significantly reduced the siRNA distribution in the other organs in mice except tumor xenograft.
